# Enhanced Emission of Fluorescein Label in Immune Complexes Provides for Rapid Homogeneous Assay of Aflatoxin B1

**DOI:** 10.3390/s25247660

**Published:** 2025-12-17

**Authors:** Dmitriy V. Sotnikov, Andrey S. Agapov, Sergei A. Eremin, Anatoly V. Zherdev, Boris B. Dzantiev

**Affiliations:** 1 A.N. Bach Institute of Biochemistry, Research Centre of Biotechnology, Russian Academy of Sciences, Leninsky Prospect 33, 119071 Moscow, Russiazherdev@inbi.ras.ru (A.V.Z.); dzantiev@inbi.ras.ru (B.B.D.); 2 Faculty of Chemistry, M.V. Lomonosov Moscow State University, Leninsky Gory 1/3, 119991 Moscow, Russia; saeremin@gmail.com

**Keywords:** fluorescein-antigen conjugates, enhanced emission, homogeneous immunoassay, control of mycotoxins, rapid tests, food safety

## Abstract

**Highlights:**

**What are the main findings?**

- Simple registration of immune interaction for labeled aflatoxin B1 was demonstrated.
- The modulation of fluorescence found was transformed into rapid and sensitive assay.

**What are the implications of the main findings?**

- Enhanced emission in immune complexes can be applied to test food samples.
- Fluorescent derivatives of antigens could be used in a new perspective assay.

**Abstract:**

Homogeneous fluorescence immunoassays are in high demand due to their simplicity, rapidity, sensitivity, and specificity. These methods typically utilize immune-induced changes in the rotational mobility of the fluorophore with depolarization of plane-polarized excitation light (FPIA, etc.) or fluorescence quenching based on intramolecular energy transfer (FRET, etc.). This article presents an immunoassay based on enhanced emission of the fluorescein label in immune complexes. Over the entire history of fluorescence immunoassay research, this effect has been described in a few papers, while it allows overcoming the limitations of prevailing approaches. We discovered the assay for detecting aflatoxin B1 (AFB1), a widespread toxic contaminant of agricultural products. The one-step assay procedure consists of mixing the sample with antibodies and fluorescently labeled AFB1, accompanied by fluorescence measurement. This method enables the detection of AFB1 at concentrations up to 200 pg/mL in 10 min, including measurements in complex samples (corn extracts). Minimal manipulations in the course of the testing also provide high accuracy. The AFB1 revealed in contaminated corn samples was in the range of 76–136%. The influence of immune complex formation on the fluorescent label’s emission can be easily tested and serve as a basis for applying this principle to other diverse analytes and various kinds of samples.

## 1. Introduction

In the modern practice of detecting toxic compounds in food products, heterogeneous methods dominate—various types of chromatography, enzyme-linked immunosorbent assay, biosensor systems and others [1,2,3]. However, homogeneous analysis methods have fundamental advantages. Direct detection of the formation of specific complexes in a solution without separating its components significantly simplifies the analysis procedure and reduces the time taken to obtain results. Moreover, unlike heterogeneous systems, the only instrument being necessary for a homogeneous analysis is a detector of the signal that was induced by a specific interaction. This simplification of the analysis process allows testing to be carried out closer to the sample collection point. However, homogeneous analysis requires the binding of the analyte to the receptor molecule (antibody, aptamer, etc.) to directly modulate the recorded signal. Since measurements are performed without removing sample components, fluorescence detection appears promising. Working with fluorescent signals, it is possible to select a fluorophore with spectral characteristics that minimize the influence of matrix components on the measurements. Existing developments of such analytical methods include polarization fluorescence analysis (PFIA) [4,5,6,7], measurements of Förster resonance energy transfer (FRET) [8,9,10], and other methods [11,12,13]. But to implement them, labeling the analytical reagent with a fluorophore is not enough; additional reagents or more complex signal recording tools are needed.

The simplest possible implementation of a homogeneous fluorescence immunoassay can be recording changes in the label’s fluorescence caused by the formation of an immune complex without additional energy acceptors. Observations of such significant changes, sufficient for analytical application, have been reported in the literature. Thus, Smith [14] described the enhancement of fluorescence of a thyroxine–fluorescein conjugate by an anti-thyroxine antiserum. He stated that iodine atoms in thyroxine quenches fluorescence, and binding to antibodies suppressed quenching. Later, it was suggested that fluorescence is enhanced upon binding to an antibody due to the transition of the fluorophore to a less polarized environment [15,16]. However, for other antigens, the opposite effect of fluorescence quenching by antibodies has been described and implemented in a row of analytical developments [17,18,19,20,21]. An advancement in understanding cases with enhanced fluorescence was made by Tan et al., who demonstrated the presence of fluorescent and non-fluorescent conformations for fluorescein-labeled Δ^9^-tetrahydrocannabinol, with binding to antibodies preventing the formation of the latter’s conformation [22]. This effect was subsequently confirmed for the interaction of thyroxine with thyroxine-binding proteins, i.e., its independence from the nature of the receptor was demonstrated [23,24]. Thus, throughout the history of fluorescence immunoassay, there are only a minimal number of observations of this effect and its application to medical analyses. Extending the applicability of this approach to a wider range of samples related to food control has not yet been considered.

We studied the application of fluorescence enhancement to the homogeneous fluorescence immunoassay of aflatoxin B1 (AFB1). Aflatoxins are a widespread group of mycotoxins, and AFB1 is considered as their most important representative for food and feed safety monitoring due to the frequency of its occurrence and toxic effects [25]. Therefore, strict regulations exist in most countries for the control of aflatoxins in agricultural products. Almost all methods currently implemented for mass food screening for AFB1 are heterogeneous. These methods have significant limitations in terms of the speed of obtaining results and the high requirements for analytical equipment and qualified personnel. Therefore, more simple and rapid methods for detecting these dangerous food contaminants are in demand.

This publication presents a study of changes in the fluorescence intensity of an AFB1-fluorescein conjugate, the development of conditions for a sensitive immunoassay based on this effect, the determination of its analytical characteristics, and an evaluation of its application to contaminated corn flour samples. The capabilities and competitive advantages of this method, as well as prospects for its further development, are also assessed.

## 2. Materials and Methods

### 2.1. Materials

Aflatoxin B1 (AFB1), Ochratoxin A (OTA), zearalenone (ZEA), T2 toxin (T2), and deoxynivalenol (DON) standards (10 μg/mL) were from the All-Russian Research Institute of Veterinary Sanitation, Hygiene, and Ecology, Moscow, Russia. Standard samples of corn flour contaminated with aflatoxins (2 ppb) and aflatoxin-free corn flour were from the Trilogy Analytical Laboratory, Washington, MO, USA. Monoclonal antibodies (MoAb) against AFB1 and against ochratoxin A (OTA) were from IL Test-Pushchino, Pushchino, Russia. Hydrochloric acid (37% solution) was from Acros Organics, Geel, Belgium; dicyclohexylcarbodiimide, N-hydroxysuccinimide, and sodium azide (99.5%) were from Sigma Aldrich, St. Louis, MO, USA. All other additional reagents were from Khimmed (Moscow, Russia). Deionized water produced by the Milli-Q system (Millipore; Burlington, MA, USA) was used to prepare working solutions.

### 2.2. Obtaining and Purification of Fluorescein-Labeled AFB1

**Figure 1:**
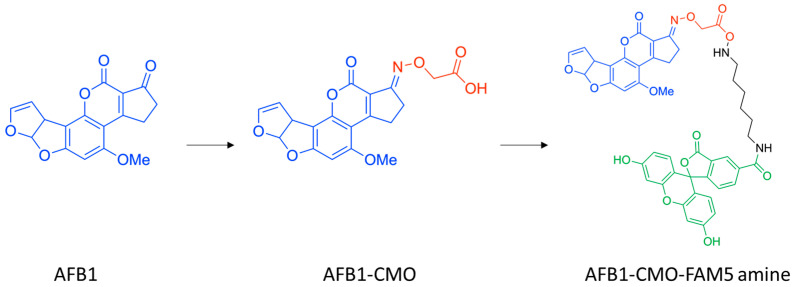
Modified compounds for obtaining fluorescently labeled AFB1.

3-(O-carboxymethyl)oxime derivative of AFB1 (Figure 1) was synthesized in accordance with the following protocol [26]: Pyridine (5 mL), AFB1 (10 mg), and carboxymethoxylamine (20 mg) were incubated for 24 h at room temperature. The pyridine was evaporated at 50 °C, and the remaining 2 mL product was dissolved in 10 mL water. Then, about 5 mL of 0.1 M NaOH was added drop-wise to reach pH = 8.0. Benzene (10 mL) was added to this solution, and the formed separated aqueous phase was acidified with 5 M HCl to pH = 2. The formed precipitate was extracted with ethyl acetate (60 mL) and dried with 2 g anhydrous Na_2_SO_4_. The organic fraction was filtered through Whatman filter paper and the solvent was evaporated.

5-Carboxyfluorescein modified by inclusion of –HN–(CH_2_)_6_–NH_2_ bridge structure (Figure 1) was purchased from Luminoprobe (Moscow, Russia).

A total of 1 mL of dicyclohexylcarbodiimide (DCC) in dimethylformamide (DMF) (50 µmol/L) and 1 mL of N-hydroxysuccinimide (NHS) in DMF (50 µmol/L) were added to 9.5 mg of AFB1-CMO (the AFB1-CMO:DCC:NHS molar ratio was 1:2.5:2.5). The mixture was stirred at room temperature overnight. Then, 2 mg of FAM-5 amine was added and the reaction mixture was kept at room temperature for 2 h with continued incubation overnight at 4 °C.

The resulting reaction mixture was purified by thin-layer chromatography (TLC) on silica sheets (10 × 10 cm; silica gel 60 with concentration zone/without fluorescence indicator, Merck (Darmstadt, Germany)). The mobile phase was MeOH-CHCl_3_ (1:3, *v*/*v*). The main yellow band, clearly visible under UV light (λ = 365 nm), was collected, dissolved in ethanol, stripped from silica via filtration through a Teflon membrane syringe filter with a pore size of 0.45 µm and 17 mm in diameter, and purified again by TLC. Ethanol as a solvent was evaporated, and the residue finally dissolved in methanol and was stored at −20 °C in the dark. Under these storage conditions, the fluorescent properties of the conjugates were stable for at least 6 months. Further in the text, the obtained derivative AFB1-CMO-FAM5 amine is designated as AFB1-5-FAM for brevity.

### 2.3. AFB1-5-FAM Analysis by HPLC-MS

The reaction products were separated by HPLC (Agilent Technologies 1200, Santa Clara, CA, USA) using a Hypersil Gold aQ 10 × 2.1 mm precolumn (3 μm particle size) and a Hypersil Gold aQ 150 × 2.1 mm analytical column (1.9 μm particle size) (Thermo Fisher Scientific, Waltham, MA, USA). Separation was carried out at 30 °C and a flow rate of 0.5 mL/min. Solvents were as follows: (a) ultrapure water/CH_3_CN, 95/5 *v*/*v*; (b) ultrapure water/HCOOH 99.9/0.1% *v*/*v*; and (c) CH_3_CN/HCOOH 99.9/0.1 *v*/*v*. The volume of the analyzed solution was 3 μL, and the analysis time was 23 min. The structure of AFB1-5-FAM was confirmed by electrospray ionization mass spectrometry on an Agilent 6460 Triple Quadrupole LC/MS system (Santa Clara, CA, USA).

### 2.4. Testing the Binding of Fluorescein-AFB1 Conjugate to Antibodies

In a black 96-well microplate, anti-AFB1 antibody was serially twofold diluted in 50 μL of 50 mM phosphate buffer, with pH 7.4, and with 0.1 M NaCl and 0.05% Tween-20 (PBST) starting from 9 μg/mL (60 nM). The AFB1-5-FAM conjugate solution in methanol was diluted in PBST to a concentration of 20 nM. A total of 50 μL of the resulting AFB1-5-FAM solution were added to the anti-AFB1 antibody dilution series. The microplate was incubated for 10 min at room temperature; the fluorescence and fluorescence anisotropy were measured by a CLARIOstar Plus multidetector (BMG Labtech, Ortenberg, Germany).

### 2.5. Competitive Immunoassay of AFB1

In a black 96-well microplate, AFB1 was serially twofold diluted in 50 μL of extracts (see Section 2.6), starting from 200 nM (62.5 ng/mL). The anti-AFB1-antibody (50 μL, 12 nM in PBST) was added to these dilutions. The microplate was incubated for 30 min at room temperature. Then, the AFB1-5-FAM solution in methanol (50 μL, 40 nM in PBST) was added to the reaction mixture after 10, 20, and 30 min, respectively. The fluorescence was measured by a CLARIOstar Plus multidetector.

The calibration curve was approximated by four-parameter sigmoidal fitting:

$$f\left(x\right)=d+\frac{a-d}{1+{\left(\frac{x}{c}\right)}^{b}}$$

Variables *a*–*d* were assigned to specific features of the curve: *a* (upper asymptote), *b* (slope at the inflection point), *c* (the inflection point; midpoints; 50% inhibitory concentration (IC50) of the calibration curve), and *d* (lower asymptote); *x* is the calibration concentration.

### 2.6. Preparation of Food Samples and Their Testing

An extract of the test sample (ground corn kernels) was prepared for analysis as follows [27]. A 1 g sample of corn kernels was weighed in a 7 mL test tube, to which 5 mL of acetonitrile was added. The mixture was incubated in a rotary mixer for 30 min at room temperature. The tube was allowed to stand for 2–5 min to allow large kernel fragments to settle into sediment. The resulting extract was withdrawn from the tube using a 12 mL syringe fitted with a 0.22 μm membrane filter. The extract was transferred to a glass weighing bottle and evaporated in a thermostat at 37 °C until the volume was less than 1 mL. Then the volume was brought up to exactly 1 mL with acetonitrile using an automatic pipette and 400 μL PBST was added. The samples were then tested as described in the previous section.

An artificial contamination of grain was carried out as follows. A fixed volume of AFB1 solution of a known concentration, calculated based on the final dilution factor during all stages of sample preparation and analysis, is added to a 1 g sample of corn grain. (After the addition of antibodies and AFB1-5-FAM conjugate, the AFB1 content in the reaction mixture becomes eight times lower than in the original contaminated grain.) Three samples of 3.2 ng AFB1 (equivalent to 0.4 ng/mL in the final solution) and three samples of 5.6 ng AFB1 (equivalent to 0.7 ng/mL in the final solution) are contaminated. The grain is thoroughly mixed and dried in an incubator at 37 °C for 60 min.

Commercial contaminated grain (Trilogy Analytical Laboratory, Washington, MO, USA) contains 2 ng of aflatoxins per gram (2 ppb). Extracts of contaminated samples, depending on the final concentration in the analyzed mixture, were prepared either from 1 g of corn kernels and 5 mL of acetonitrile (0.25 ng/mL from a commercial sample, 0.4 ng/mL and 0.7 ng/mL from in-house samples) or from 2 g of corn kernels and 10 mL of acetonitrile (0.5 ng/mL from a commercial sample, 0.8 ng/mL, and 1.4 ng/mL from in-house samples) using the same method as extracts of clean grain of the corresponding mass.

Data for contaminated samples were obtained using the same procedure as the calibration curve, but with modified sample preparation. Eight points are measured in two replicates: six obtained extracts (final concentrations of 0.25; 0.4; 0.5; 0.7; 0.8; 1.4 ng/mL AFB1), and were prepared without antibodies or AFB1 (50 μL of PBST was added instead of them).

## 3. Results and Discussion

### 3.1. Synthesis and Characterization of AFB1-5-FAM Conjugate

To synthesize the AFB1-5-FAM conjugate, a carboxymethyloxime-AFB1 derivative with a molecular weight of 385 Da was used, which was coupled to 5-carboxyfluorescein modified by –HN–(CH_2_)_6_–NH_2_ bridge with a total molecular weight of 475 Da. According to HPLC-MS, the reaction product gives a major peak in the positive ion mode at 842, which corresponds to the molecular weight of the final reaction product shown in the scheme in Figure 1. This confirms the success of the conjugate synthesis.

The final product was purified from unreacted hapten and labeled using thin-layer chromatography (TLC). Repeated TLC separation revealed no additional bands, indicating the purity of the resulting product.

### 3.2. Influence of Antibodies on Conjugate Fluorescence

**Figure 2:**
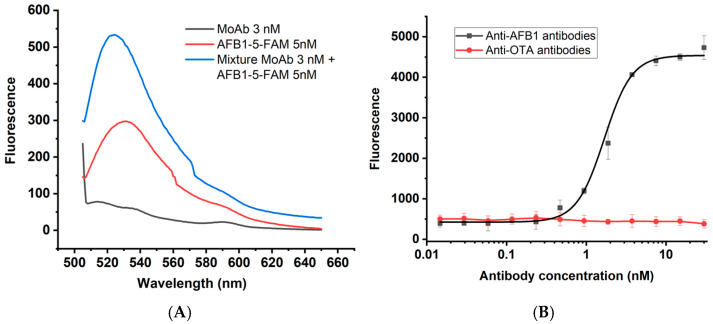
Effect of antibodies on AFB1-5-FAM fluorescence. (**A**) Fluorescence spectra. (**B**) AFB1-5-FAM fluorescence in the presence of anti-AFB1 antibodies and anti-OTA antibodies.

We were interested in the fluorescence of the conjugate in its free form and in its antibody-bound form. Therefore, we recorded fluorescence spectra for AFB1-5-FAM, anti-fluorescein antibodies, and their mixture at the peak excitation of 5-FAM. Figure 2A shows the fluorescence spectra for the antibodies (3 nM), conjugate (5 nM), and their mixture when irradiated at the 5-FAM excitation wavelength of 492 nm. Given the bivalent nature of the antibodies, the concentration of paratopes in the solution slightly exceeds the concentration of conjugate.

When testing antibody-bound conjugate, we found that complex formation approximately doubled the absolute fluorescence value, as shown in Figure 2A. Due to the limited data available, the mechanism behind this phenomenon is controversial. Various hypotheses have been put forward, including intramolecular quenching of the label by the hapten itself, which is suppressed upon binding to antibodies, and changes in the molecular environment of the label upon binding to antibodies [15]. It can also be suggested that the quenching is caused by contact with certain functional groups of amino acids in antigen-binding site of antibodies.

As seen from the spectra, the formation of the immune complex also significantly shifts the tracer fluorescence peak: from 532 nm for the pure conjugate to 524 nm for its complex with antibodies. That is, the fluorescence of the bound conjugate becomes closer to the spectrum of pure 5-FAM, with an emission maximum at 518 nm [28]. This supports an intramolecular mechanism of 5-FAM quenching by the hapten, which is suppressed upon hapten binding to antibodies, resulting in the fluorescent properties of the label becoming closer to those of the original fluorophore.

Figure 2B shows fluorescence dependence at 524 nm of the antibody–tracer mixture at different antibody concentrations. Anti-OTA antibodies were used as a negative control to confirm the specificity of the interaction. Based on the obtained dependence of the effect of different antibody concentrations on conjugate fluorescence, the antibody concentration for the competitive assay was selected. The antibody concentration chosen for more effective competition, according to existing recommendations [29,30], corresponded to 70% of the upper plateau signal and was 3 nM.

Based on the titration curve of specific antibodies given in Figure 2B, the equilibrium interaction constant was calculated according to the method described in [31]. The obtained Kd was 7 × 10^−10^ M. This value allows estimating the theoretical limit of hapten detection in the competitive assay, since the IC50 value of the calibration curve tends to Kd with decreasing concentrations of interacting reagents in the reaction mixture [32].

### 3.3. Competitive Immunofluorescence Assay

Since fluorescence depends on the reaction medium, it is important to understand the suitability of the method for real-world multicomponent samples. We analyzed grain samples after aflatoxin extraction with acetonitrile.

**Figure 3:**
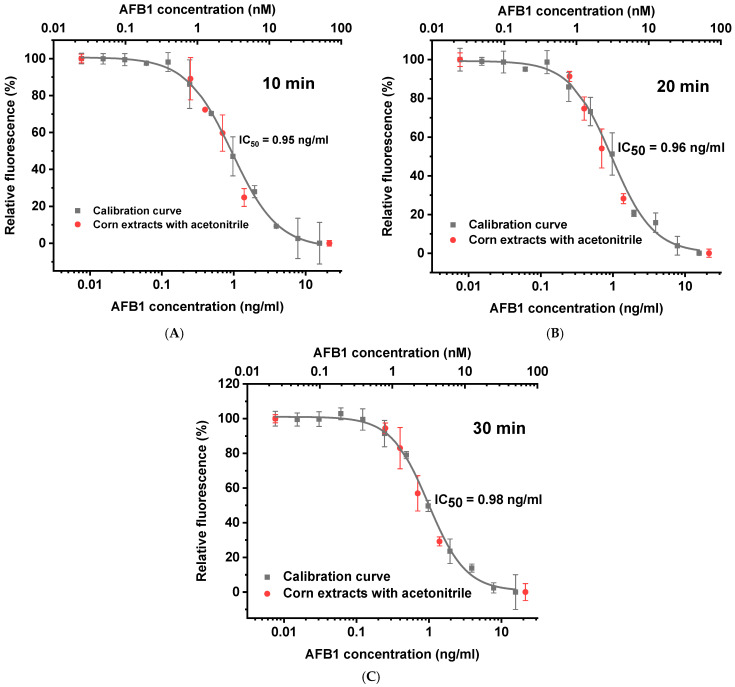
Competitive homogeneous fluorescence-enhanced immunoassay for 10 (**A**), 20 (**B**), and 30 (**C**) min of reactants incubation. Black curves represent calibration curves obtained in standard corn extracts. Red dots represent artificially contaminated corn samples containing 21.4, 1.4, 0.7, 0.4, 0.25, and 0.0076 ng/g AFB1.

The concentration values obtained 10, 20, and 30 min after mixing the reagents are shown as black curves in Figure 3. As seen from the graphs, the position of the calibration curve (see IC50) changes little over the 10–30 min interval. The detection limit of AFB1 was 200 pg/mL. The detection limit was defined according to IUPAC recommendations as the analyte concentration, the signal for which exceeds the signal for the negative sample by three standard deviations of the signal for the negative sample.

The IC50 values for the calibration curves are near 3 nM, or approximately four times the Kd value. This means that, if necessary, the assay sensitivity can be increased by reducing the concentrations of antibodies and labeled conjugate, but this will also increase the error due to a decrease in value of registered signals.

The obtained dependences demonstrate high efficiency in detecting AFB1 in contaminated corn samples without significant losses during sample preparation or due to matrix effects. Further testing of extracts of artificially contaminated grain showed (red dots in Figure 3) that in all cases, the deviation in the toxin content determined using calibration curves differed from the initial content by no more than 36%. The quantitative results for detecting AFB1 are summarized in Table 1. For quantitative determination, concentrations of 0.25, 0.4, 0.7, and 1.4 ng/mL were taken, which are within the working range of the calibration curves in Figure 3.

### 3.4. Specificity of AFB1 Detection by the Developed Assay

**Figure 4:**
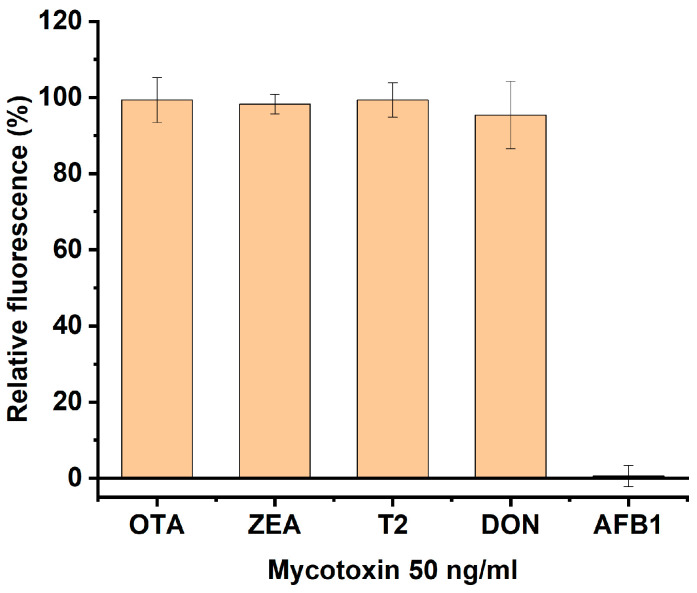
Detection of mycotoxins in a homogeneous fluorescence immunoassay in corn extracts spiked with 50 ng/mL of ochratoxin A (OTA), zearalenone (ZEA), T2 toxin (T2), deoxynivalenol (DON), and aflatoxin B1 (AFB1).

To confirm the specificity of the system’s response to AFB1, experiments were conducted on corn extracts contaminated with other mycotoxins. AFB1, OTA, ZEA, T2, and DON were added to the extracts at concentrations of 50 ng/mL. Figure 4 demonstrates that for AFB1 addition, the fluorescence decreased to the level of the free AFB1-5-FAM conjugate. For other mycotoxins, fluorescence remained the same as for the AFB1-5-FAM conjugate bound with the antibodies. Thus, the assay demonstrated the absences of responses to other mycotoxins, confirming its high specificity.

### 3.5. Comparison with Other Homogeneous Immunoassays of AFB1 and Practical Recommendations

Modern analytical methods used for mass screening should be not only highly accurate and sensitive but also cost-effective and highly productive. Otherwise, their use will incur unreasonable costs. Homogeneous assays based on antibody-induced modulation of fluorescence of a fluorescent label fully satisfy all these requirements [15,16]. However, this assay format is currently rarely used in practice. This is because its applicability is determined by the properties of the labeled hapten. For efficient assay, the binding of the hapten–label conjugate to antibody should result in a significant change in fluorescence intensity. To date, successful implementations of this approach have been described for a limited number of compounds [8,14,17,18,19,20,21,22].

Our study demonstrated for the first time that aflatoxin B1, a major food contaminant, possesses the necessary properties for a homogeneous enhanced immunofluorescence assay. The developed method is characterized by high accuracy due to minimal manipulation, as well as high sensitivity due to the reaction being conducted in a homogeneous phase and the absence of diffusion-limiting factors [33]. At the same time, the method enables results to be obtained in minimal time: in fact, the most time-consuming step is extraction, i.e., sample preparation, while the analysis itself can be completed in 10 min. Moreover, the measurement technique is simpler than for FPIA [26], as it does not require the use of polarizers, but only direct measurement of fluorescence intensity. The necessary reagents are unmodified antibodies and a fluorescently labeled antigen, unlike the FRET method [9], which requires labeling both components of the immune pair with a label and quencher.

The reached sensitivity of the assay is comparable or superior compared with earlier described homogeneous immunoassays of AFB1, such as 0.95 ng/mL in [33], 2.6, 0.4, and 0.6 ng/mL for three assay formats in [34], 2.3 nM (0.717 ng/mL) in [35], and 0.85 (intact antibody) and 0.09 ng/mL (Fab fragment) in [8].

Permissible concentrations of AFB1 in food products vary among countries [36]. The most stringent requirements are adopted in the European Union: the AFB1 content in products (except for baby food) should not exceed 2–8 μg/kg (ng/g) depending on the product [37]. This means that the method has a 10-fold sensitivity margin relative to the most stringent AFB1 MRL requirements.

The additional gain in sensitivity can be used, for example, to dilute the sample further in order to reduce the influence of the matrix effect on the analytical results. According to the sample preparation method used, the AFB1 present in the grain is diluted eightfold, which corresponds to a detection limit of 1.6 ng/g based on the initial AFB1 content in the grain. If necessary, lower AFB1 concentrations can be detected, for example, by increasing the extract evaporation time.

## 4. Conclusions

The proposed method is characterized by methodical simplicity and high sensitivity. Conducting homogeneous analysis allows for the reduction of time taken to obtain results and excludes the stages of separation of bound and free reagents, which is typical for heterogeneous immunoassays, such as ELISA. Measuring fluorescence requires simpler and cheaper equipment than fluorescence polarization in the case of FPIA.

## Figures and Tables

**Table 1:** Detection of AFB1 in corn extracts using the developed method.

Added AFB1 Concentration, ng/mL	Detected AFB1 Concentration, ng/mL /Degree of Revealing, %		
	10 min	20 min	30 min
0.25	0.21/84	0.19/76	0.21/84
0.40	0.45/113	0.46/115	0.40/100
0.70	0.70/100	0.84/120	0.85/121
1.40	1.90/136	1.75/125	1.68/120

## Data Availability

The original contributions presented in this study are included in the article. Further inquiries can be directed to the corresponding author.
